# Iron Deficiency Anemia among In-School Adolescent Girls in Rural Area of Bahir Dar City Administration, North West Ethiopia

**DOI:** 10.1155/2019/1097547

**Published:** 2019-03-21

**Authors:** Getachew Mengistu, Muluken Azage, Hordofa Gutema

**Affiliations:** ^1^Department of Regulatory, Bahir Dar City Administration Health Office, Post Code 50, Ethiopia; ^2^Department of Environmental Health, School of Public Health, College of Medicine and Health Sciences, Bahir Dar University, Post Code 79, Ethiopia; ^3^Department of Health Promotion and Behavioral Sciences, School of Public Health, College of Medicine and Health Sciences, Bahir Dar University, Post Code 79, Ethiopia

## Abstract

**Background:**

Anemia is a major public health problem worldwide. Adolescent girls are the most vulnerable group of population due to different reasons. The aim of this study was to assess the prevalence of anemia and associated factors among school adolescent girls in rural towns of Bahir Dar City Administration, North West Ethiopia.

**Methods:**

A cross-sectional study was conducted from March 5 to April 15, 2017, on 443 randomly selected school adolescent girls. Data were collected using pretested structured questionnaire and anthropometric measurements. Blood sample was also collected to assess the hemoglobin (Hgb) value of study participants. SPSS version 20 was used to analyze data. Descriptive statistics were used to describe data. Bivariate and multivariable logistic regression models were used to identify the associated factors with the outcome variable. Crude and adjusted odds ratios with 95% confidence interval (CI) were calculated to identify the variables significantly associated with the outcome variable.

**Result:**

The prevalence of anemia was 11.1%. Household family size* [AOR=3.2, 95%CI (1.29-7.89)]*, average household monthly income <500 ETB* [AOR=10; 95%CI (2.49-41.26)]*, 501-1000 ETB* [AOR=6, 95%CI (2.54-14.33)]*, history of intestinal parasitic infection* [AOR=2.7; 95% CI (1.19-6.21)]*, duration of menstruation flow* [AOR=2.4; 95%CI (1.08- 5.44)]*, and BMI for age* [AOR-3.2; 95% CI (1.43-7.05)]* were the predictors of anemia.

**Conclusion and Recommendation:**

Anemia was a mild public health problem among school adolescent girls in the study area. Household monthly income, family size, intestinal parasite infections, duration of menstruation, and BMI for age are predictors of anemia. Thus, intervention strategies should focus on prevention and early treatment of intestinal parasite, nutritional education, screening, and iron supplementation programs to prevent anemia among school adolescent girls.

## 1. Introduction

Anemia is defined as a condition in which the number of red blood cells (RBCs) and their oxygen-carrying capacity is insufficient to meet the body's physiologic needs. It is a condition when the normal number of RBCs (<4.2 million/*μ*l) or hemoglobin (Hb) level <12 g/dl) in women and < 13 in men [1]. Globally, anemia is the most common and inflexible nutritional problem affecting around 2 billion of the world's population having major impact on human health and social and economic development; and more than 89% of this burden occurred in developing countries [2, 3].

Accounting half of all cases, iron deficiency anemia is the most common cause of anemia. However, other conditions like nutritional deficiencies, acute and chronic inflammation, parasitic infections, growth spurt, increase in iron requirements, increased iron loss from the body during the menstruation, inherited or acquired disorders of hemoglobin synthesis, RBC production, or survival are also considered cause of anemia [1].

Even though iron deficiency anemia can possibly occur at all stages of the life, it is more prevalent among pregnant women, young children, and adolescents. Since the overall iron requirement increases two- to threefolds during adolescence due to high growth spurt and the loss of 12.5-15 mg iron each month, adolescent girls are vulnerable to anemia. Anemia during adolescence is nutritional problem and it has irreversible negative effects on growth and cognitive, work performance and serious impact throughout the reproductive years of life and beyond. Occurrence of pregnancy during adolescence with anemia increases not only the maternal morbidity and mortality but also the incidence of poor maternal birth outcomes such as still birth, low birth weight, and prematurity and also has negative impact on infant iron status [2, 4–6].

The impact of anemia among adolescent girls is still public health problem globally although there are specific actions like encouraging consumption of iron-rich foods through dietary change, nutritional education, treatment and prevention of parasitic infections, weekly iron supplementation to prevent iron-deficiency anemia, and improving iron status among adolescent girls [2, 5].

Different researchers have conducted studies on anemia among adolescent girls from different part of the world. However, the age range which these scholars considered as adolescent differs among the studies and they were not the standard age category between 10 and 19 years. Since using the finding of studies that use different age ranges can negatively affect the impact of interventions, we argue that the studies should be conducted by selecting the appropriate age group [7–11].

Indeed, the government of Ethiopia had implemented the National Nutrition Program II focusing on reducing prevalence of stunting, wasting, and chronic undernutrition among women of childbearing age. However, intermittent weekly oral iron supplementation for children which is recommended by the WHO was not practically implemented yet to prevent the risk of iron deficiency anemia during childhood [2, 12]. Additionally, studies conducted on anemia among the adolescent girls in the country are not only few in number but also did not address these adolescents' living conditions and their knowledge on anemia prevention and food rich of iron and the effect that these variables have on anemia. In the current study, these factors were considered [11, 13–15].

Furthermore, determining the prevalence of iron deficiency anemia and those factors associated with it among adolescent girls is crucial for initiation of effective intervention that improve their nutritional status to prevent occurrence of different risks during their adolescence, pregnancy, child birth, and beyond. Therefore, the aim of this study is to assess the prevalence of anemia and associated factors among school adolescent girls in rural areas of Bahir Dar City Administration, North West Ethiopia.

## 2. Materials and Methods

### 2.1. Study Design and Period

A cross-sectional study was conducted from March 5 to April 15, 2017, to assess the prevalence of anemia and associated factors among school adolescent girls.

### 2.2. Study Area and Population

The study was conducted at rural towns of Bahir Dar city administration. Bahir Dar city is located in Amhara region North West Ethiopia which is 565 KMs from Addis Ababa, capital city of Ethiopia. Meshenti, Tiss Abay, Zegie, and Zenzelema are the four rural towns in Bahir Dar city administration. In these rural towns, there are 4 elementary and 3 secondary schools. School girls aged between 10 and 19 years in rural towns of Bahir Dar city administration were the study population. Adolescent girls who were critically ill at the time of data collection and pregnant were excluded from the study.

### 2.3. Sample Size and Sampling Procedure

The sample size was calculated using Epi info version 7 open software by considering variables that determine anemia in previous study [11]. The final adequate sample size (443 adolescent girls) was obtained using the following parameters: odds ratio of consumption of fruit (5.1), 95% CI, and power 85%. Sampling frame was prepared using the school roster sheets. Proportion to size allocation was made to determine the required sample size from each school. Simple random sampling technique was used to select study participants from the registration roster sheet.

### 2.4. Data Collection Tools and Procedures

Data were collected using pretested structured questionnaire. The questionnaire had sociodemographic characteristics, knowledge questions, dietary practice, health, and nutritional status. The English version questionnaire was translated in to national language (Amharic) and then back to English to check its consistency. Those school adolescent girls who were absent at the time of data collection were followed and recontacted for the next additional 1 to 2 days to include in this study. Three diploma nurses and one diploma laboratory technician were recruited and trained for data collection. One-degree holder nurse was recruited as supervisor during data collection.

### 2.5. Measurements

*Outcome Variables*. Anemia was the outcome variable of the study. If the level Hb is <12 g/dl in adolescent girls, it indicates the presence of anemia. Hb level between 11 to 11.9 g/dl and 8 to 10.9 g/dl indicates mild and moderate anemia, respectively, while Hb level of <8.0 g/dl is indication of severe anemia in adolescent girls [16].

*Independent Variables*. These are sociodemographic characteristics, knowledge on anemia, dietary practice, health, and nutrition condition. Sociodemographic characteristics include adolescent's age, education level, place of residence, and occupation of her parents/guardian. Knowledge on anemia was measured using ten items by summing the statement related to anemia, its common cause, sign and symptoms of anemia, consequences, treatment, and prevention after reversely scoring the incorrect statements. Dietary diversity practice was measured using the number of different food groups irrespective of the amount consumed over the past 24-hour period.

Health and nutrition condition include menstrual status, history of parasitic and malaria infection, and iron supplementation.

Body mass index (BMI) was calculated as weight for height (BMI for age) using WHO anthroplus software. The weight was measured by wearing light clothing to the nearest 0.1 kg by standing in the center of the platform of the scale and remaining motionless until the measurement can be obtained. The height was measured using a wooden height measuring board with a sliding head bar to the nearest 0.1 cm on standing Frankfurt position without shoes. Finally, BMI for age of ≥-2SD and <-2SD were considered normal and underweight or thinness, respectively.

*Dietary Diversity Score (DDS)*. The DDS was calculated by summing the number of different food groups irrespective of the amount consumed over the past 24-hour period. It was classified as poor, medium, and high DDS if the participants consume 3, 4 – 6, and > 6 food items in the last 24 hours, respectively.

### 2.6. Data Quality Assurance

Training was given to the data collectors and supervisors on the data collection procedures and techniques. Before actual data collection, the questionnaire was pretested in school which is outside of study area and modification on some questions was made to increase the clarity of questions. The filled questionnaire was reviewed and checked on a daily basis for completeness and consistency by the supervisors and the principal investigator.

### 2.7. Data Processing and Analysis

Data was checked, cleaned, coded, and entered using Epi info version 7 and then exported to SPSS version 20 statistical software for analysis. Descriptive statistics such as frequencies, proportions, and standard deviation were done to describe data. Bivariate logistic regression analysis was done to check association between each independent variable with the outcome variable (anemia). All variables with p-value < 0.2 in the bivariate analysis were entered into the multivariable logistic regression analysis. Adjusted odds ratio (AOR) with 95% confidence interval (CI) was used to identify the independent predictors of anemia. A p-value <0.05 was set as cut-off value for statistical significance.

## 3. Results

### 3.1. Sociodemographic Characteristics of Study Participants

A total of 423 school adolescent girls participated in this study, making response rate 95.5%. The mean (±SD) age of study participants was 14.5 (±2.28) years. Above ninety percent of the study participants (94.3%) were Orthodox Christianity followers. Three hundred and two (71.4%) and 267(63.1%) of the study participants' fathers and mother's occupation were farmers and house wives, respectively. Regarding the educational status, 228 (53.9%) and 156 (36.9%) of mothers/guardians and fathers/guardians were unable to read and write, respectively. Around one-third (30.3%) of study participants had an estimated average monthly household income of less than 1000 Ethiopian birr. Almost all 416 (98.3%) of study participants lived with their parents, of which 392 (92.7%) of adolescent girls lived with both father and mother (Table 1).

### 3.2. Knowledge of Study Participants about Anemia

More than three-fourths, 332 (78.5%), of the participants had not heard about anemia. Majority 240 (56.7%) of participants had poor knowledge on anemia. Around 43.3% adolescent girls had good knowledge about anemia. Of all study participants, only 162 (38.3%) had good knowledge on the causes of anemia, 178 (42%) on signs and symptoms of anemia, 196 (46.3%) on consequences of anemia, and 183 (38.5%) on prevention of anemia (Table 2).

### 3.3. Dietary Diversity Score (DDS) and Practice

Less than half (46.1%) of study participants had a medium DDS. Majority, 349 (82.5%), of adolescent girls were reported that they consume bread and rice, pasta, biscuits, or any other foods made from cereals followed by consumption of fats and oils 331(78.3%) and roots and tubers 285 (67.4%). Majority (85.2%) of the respondents had meal frequency of three or more times per day. More than half 226 (53.4%) of the respondents said they drink tea immediately after meal within 30 minutes. Only 32 (9.2%) of the respondents ate fruits three or more times per week. Only few (0.7%) of the study participants reported that there are foods that are restricted for adolescent girls within the community. Almost all 419 (99%) of them did not have a history of iron supplementation (Table 3).

### 3.4. Prevalence of Anemia, Health, and Nutritional Status

The prevalence of anemia was found to be 47 (11.1%) of which 46 (97.8%) of them had mild anemia. Above half (54.8 %) of study participants were thin. Nine in ten (91.7%) and eight in ten (80.9%) of the study participants have no history of malaria and intestinal parasite infection in the last one month, respectively. More than two-thirds (67.7%) of adolescent girls had already menstruated. Around 44 % of adolescent girls had duration of menstrual flow ≥ 5 days during each cycle (Table 4).

### 3.5. Factors Associated with Anemia among Adolescent Girls

In the bivariate analysis variables like age, average household monthly income, household family size, post meal consumption of tea, history of intestinal parasite infection, duration of menses, and BMI status of adolescent girls were significantly associated with anemia (p-value <0.2) and were entered to multivariable analysis.

In multivariable logistic regression analysis, average household monthly income, household family size, duration of menstrual flow per each cycle, history of intestinal parasite infection, and BMI for age were significantly associated with anemia. Adolescent girls who had household family size of > 5 were 3.2 times more likely to be anemic, compared with those who had household family size of ≤5 [*AOR =3.2, 95% CI (1.29, 7.89*)]. Adolescent girls whose family average monthly income is <500 were 10 and 6 times more likely to be anemic as compared to those whom family average monthly income 501-1000 and >1000 ETB with [*AOR=10, 95% CI (2.49, 41.26)*] and [*AOR=6, 95% CI (2.54, 14.33)*], respectively.

Adolescent girls who had history of intestinal parasite in the last one month were 2.7 times [*AOR= 2.7, 95% CI (1.19, 6.21)*] more likely to develop anemia compared with those without history of intestinal parasite infection. Adolescent girls who experience menstrual flow ≥5 days per each cycle were 2.4 times* [AOR=2.4, 95 CI% (1.08, 5.44)]* more likely to develop anemia compared to those with who experience it < 5 days. Similarly, adolescent girls with BMI for age < -2 SD were 3.2 times [*AOR=3.2, 95% CI (1.43, 7.05*) more likely to be anemic compared with those BMI for age ≥ -2 SD. (Table 5).

## 4. Discussion

In the present study, the prevalence of anemia was found to be 11.1%; out of this (95.8%) and (2.2%) had mild and moderate anemia, respectively. The finding of this study is comparable with a study conducted in Kebena district (12%) [15]. But it was lower than those similar studies reported from different areas in Ethiopia like it was 22.8% in Berahle district of Afar region and 32% in Babile district of East Harerge, Eastern Ethiopia [11, 13]. The possible reasons for this variation might be due to low socioeconomic status, small meal frequency, and hunger at those other study areas.

The finding of this study was also lower than similar studies conducted in Yala division, Siaya district of Kenya (26.5%), and western Kenya 19.8%, respectively [9, 10]. The possible reason for this variation might be due to high malaria and intestinal parasite infection, excessive heavy, and irregular menstruation at those study settings. Similarly, it was also lower than those studies reported from Karad district Satara of Maharashtra (45.3%), Lucknow district, Ultarpradesh of India (88.3%) and Anganwadi, Hassan district (45.2%), and Central Kerala of India (21%), respectively [16–19]. This may be due to irregular, prolonged and heavy amount of menstruation, high intestinal parasite infections and undernutrition, poor knowledge on nutrition and anemia, and skipping of meals.

School adolescent girls who had family size of > 5 were 3.2 times more likely to be anemic as compared to those school adolescent girls with a family size of <5. This finding was in agreement with the finding which is reported by a study done in Bonga town, southwest Ethiopia; household family size ≥5 was 2.58 times more likely to develop anemia as compared to household family size <5 [14]. It was also in line with a study conducted in Tang ail region of Bangladesh, Guntur, Andhra Pradesh of India, Khordha Rural District of Odisha in India, and Chennai, Tamil Nadu of India [8, 20–22]. This might be due to the reason that the large size of the family can be related to low care for each family member and low family income to obtain variety of foods rich in iron and other micronutrients in those study areas.

Adolescent girls whose average household income is less than 500 ETB were 10 times more likely to develop anemia and those adolescent girls with 501-1000 ETB were 6 times more likely to develop anemia. This was consistent with the finding reported in Berahle district of Afar region that low socioeconomic status was 2.8 times more likely to develop anemia compared to medium ones [11]. This finding was also comparable with studies conducted in Tang ail region of Bangladesh, Guntur, Andhra Pradesh of India, Khordha rural district, Odisha of India, and Hassan District of south India [8, 20, 21, 23]. This might be due to low family income to obtain variety of foods rich in iron and there should be a means in which iron supplementation started for adolescent girls.

Adolescent girls who had history of intestinal parasite were 2.7 times more likely to be anemic compared to those who did not. It was in agreement with a study done in Yala division, Siaya District of Kenya, in which respondents who tested positive for ova of Ascaris were 8 times more likely to develop anemia compared to those who tested negative [9]. This finding was consistent with a previous similar study conducted in Bonga Town, southwest Ethiopia; adolescents who had intestinal parasitic infection were 5.37 times more likely to develop anemia compared to those who did not [14]. Although we were not sure which specific intestinal parasite infection the girls had, intestinal parasites have their own contribution to blood loss which further lead to anemia.

Adolescent girls who had menstrual flow for ≥ 5 days were 2.4 times more likely to be anemic as compared to those adolescent girls with menstrual flow < 5 days per each cycle. This finding was in agreement with similar findings reported in Tang ail region of Bangladesh, Guntur, Andhra Pradesh of India, Khordha rural district, Odisha of India, and Western Kenya [8, 10, 20, 21]. This may due to the fact of blood loss during the menstruation.

Anemia is significantly associated with low BMI for age. Adolescent girls who had a low BMI for age were 3.2 times more likely to be anemic as compared to those who have a BMI for age. Similar findings were also reported in Bonga Town; those with low BMI for age were 2.54 times more likely to develop anemia compared to those with high BMI for age [14]. It was also parallel with the finding in Tangail region of Bangladesh and Chennai, Tamil Nadu of India [20, 22]. Since this study used cross-sectional design the cause-effect relationship was not addressed. Additionally, the study period which is fasting time might have affected the real dietary diversity practice of school adolescent girls.

## 5. Conclusions

Anemia was found to be a mild public health problem in the study area. Household monthly income, family size, intestinal parasite infections, duration of menstrual flow per each cycle, and BMI for age were the main predictors of anemia. Thus, school-based Iron folic acid supplementation and regular nutritional screening and deworming program should be implemented to help adolescent girls who are at risk of anemia.

## Figures and Tables

**Table 1:** Sociodemographic characteristics of school adolescent girls at rural towns of Bahir Dar city Administration, 2017(n=423).

*Variables*	Categories	frequency	Percentage
Age of the respondent	10-14	223	52.7
	15-19	200	47.3
Grade of the respondent	3-8	292	69
	9-10	131	31
Residence of the respondent	Urban	179	42.3
	Rural	244	57.7
Religion of the respondent	Orthodox Christian	399	94.3
	Muslim	22	5.2
	Protestant	2	0.5
Ethnicity of the respondent	Amhara	419	99
	Others	4	1
Educational status of mother/guardian/	Unable to read and write	228	53.9
	Able to read and write	98	23.2
	Elementary education	83	19.6
	Secondary and above	14	3.3
Educational status of father/guardian/	Unable to read and write	156	36.9
	Able to read and write	119	28.1
	Elementary education	112	26.5
	Secondary and above	36	8.5
Occupational status of father /guardian	Farmer	302	71.4
	Merchant	88	20.8
	Civil servant	20	4.7
	Daily worker	13	3
Occupational status of mother /guardian	House wife	267	63.1
	Merchant	82	19.4
	Farmer	45	10.6
	Civil servant	16	3.8
	Daily worker	13	3.1
House hold family size	≤ 5	210	49.6
	>5	213	50.4
Average monthly income in ETB	<500	24	5.6
	501- 1000	150	35.4
	>1000	249	59
Marital status of the respondent	Married	7	1.7
	Single	416	98.3
Living status of parent	Both are alive	392	92.7
	Only one alive	28	6.6
	Both are not alive	3	0.7
Living condition with the family	With the family	416	98.3
	With other blood related	7	1.7

**Table 2:** Knowledge about Anemia among school adolescent girls at rural towns' elementary and secondary schools of Bahir Dar city Administration, 2017 (n=423).

Knowledge about anemia	Frequency	Percentage
*Knowledge about the cause of anemia *		
Poor	261	61.7
Good	162	38.3
*Knowledge about sign and symptoms of anemia *		
Poor)	245	58%
Good	178	42%
*Knowledge about consequences of anemia *		
Poor	227	53.7%
Good	196	46.3%
*Knowledge about prevention of anemia *		
Poor	260	61.5%
Good	163	38.5%
*Over all knowledge on anemia *		
*Poor*	240	56.7%
*Good *	183	43.3%

**Table 3:** Dietary diversity score (DDS) and practice of school adolescent girls at rural towns of Bahir Dar city Administration. 2017 (n=423).

Variables	Frequency	Percent
*Dietary diversity score*		
0-3 food items (Poor DDS)	117	27.7
4- 6 food items (Medium DDS)	195	46.1
> 6 food items (Good DDS)	111	26.2
*Group of food items consumed in the last 24 hours*		
Bread and other cereals	349	82.5
Green leafy Vegetables	234	55.3
Fruits	134	31.7
Roots and tubers (like potatoes)	285	67.4
Meat, poultry& fish	44	10.4
Legumes (Bean, pea, lentils or nuts)	205	48.5
Egg	36	8.5
Milk and milk products	64	15.1
Fats and oils	331	78.3
Foods containing fat and sugar	228	53.9
*Meal frequency per day*		
Twice	57	13.5
Three and more	361	85.3
*Post meal consumption of Tea (within 30 min)*		
No	226	53.4
Yes	197	46.6
*Post meal consumption of Tea (within 30 min)*		
Always	43	21.8
Sometimes	154	78.2
*Post meal consumption of Coffee (within 30 min)*		
No	233	55
Yes	190	45
*Post meal consumption of Coffee (within 30 min)*		
Always	63	33
Sometimes	127	67
*Consumption of green leafy vegetables*		
None	132	31.2
1-2 times/week	252	59.6
>=3 times/week	39	9.2
*Free school feeding program*		
No	423	100
Yes	0	0
*Food restriction*		
No	420	99.3
Yes	3	0.7
*History of iron supplementation*		
No	419	99
Yes	4	1

**Table 4:** Anemia, health, and nutritional status of school adolescent girls at rural towns of Bahir Dar city administration, 2017 (n=423).

Variables	Frequency	Percentage
*Status of menarche*		
Not attained	141	33.3
Attained	282	67.7
*Duration of blood flow per each menses*		
<5days	*158*	*56*
≥5days	*124*	*44*
*History malaria infection*		
No	388	91.7
Yes	35	8.3
*History of intestinal parasite infection*		
No	342	80.9
Yes	81	9.1
*BMI for age (<-2 SD)*		
Thin	187	44.2
Normal	232	54.8
Over weight	4	1
*Prevalence of anemia*		
Yes	47	11.1
No	376	88.9
*Degree of anemia among anemic cases*		
Mild anemia	46	97.8
Moderate anemia	1	2.2

**Table 5:** Factors associated with anemia among school adolescent girls at rural town of Bahir Dar city administration. 2017(n=423).

Variable	Anemia		COR (95% CI)	AOR (95% CI)
	Yes (%)	No (%)		
Household family size				
≤5	15(7)	195(93)	1	1
>5	32(15)	181(85)	2.3(1.20,4.38)	*3.2 (1.29, 7.89) ∗*
Average monthly income in ETB				
< 500 ETB	6(25)	18(75)	6.58(2.21,19.59)	*10 (2.49,41.26) ∗*
501-1000 ETB	29(19.3)	121(80.7)	4.73(2.33.9.60)	*6 (2.54,14.33) ∗*
>1000 ETB	12(4.8)	237(95.2)	1	1
History of worm infestation				
No	28(8.3)	311(91.7)	1	1
Yes	19(22.6)	65(77.4)	3.25(1.71,6.16)	*2.7(1.19,6.21) ∗*
Duration of blood flow in each menses				
<5days	12 (7.6)	146(92.4)	1	1
≥5days	26 (21)	98(89)	3.23(1.56,6.75)	*2.4 (1.08,5.44)∗*
BMI for age				
Thin	30 (15.8)	159(84.2)	2.41(1.28,4.52)	*3.2(1.43,7.05)∗*
Normal	17 (7.3)	217(92.7)	1	1

COR = crude odds ratio; AOR= adjusted odds ratio; CI = confidence interval.

ETB = Ethiopian Birr; BMI = body mass index; ∗= significant at P value < 0.05.

## Data Availability

The data used to support the findings of this study are available from the corresponding author upon request.
